# Development and Validation of a Cannabinoid Quantification Method in Oil and Marijuana by UHPLC-MS

**DOI:** 10.1021/acsomega.5c07689

**Published:** 2025-11-18

**Authors:** João Victor M. de Almeida, Nathália S. Conceição, Alan Reinke Pereira, Marcos Valério V. Lyrio, Rafael S. Ortiz, Nayara A. dos Santos, Wanderson Romão

**Affiliations:** a Chemistry Department, 28126 Federal University of Espírito Santo, Vitória, Espírito Santo 29075-910, Brazil; b Instituto Nacional de Ciência e Tecnologia Forense (INCT Forense), Porto Alegre 90650-001,Brazil; c Brazilian Federal Police, Regional Superintendence of Rio Grande do Sul, Porto Alegre, Rio Grande do Sul 90160-093, Brazil; d Federal Institute of Espírito Santo, Vila Velha, Espírito Santo 29106-010, Brazil

## Abstract

*Cannabis sativa* L. is an ancient species that has been cultivated over the years for various applications, including recreational and medicinal use. Nowadays, the demand for its products has grown significantly, leading to an increase in the search for medicinal oils and a rise in smuggling, making it the main trafficked drug. In this context, our work optimized the extraction process of six cannabinoids in *Cannabis* oil and marijuana samples, as well as an analytical validation of a quantitative and qualitative method for seven cannabinoids using ultra-performance liquid chromatography coupled with low-resolution mass spectrometry (UHPLC-LTQ-MS). The optimization showed that ethyl acetate is the most suitable solvent for oil extraction. For the marijuana sample, the extraction was performed using the same solvent and sonication and vortex agitation for 10 and 7.5 min, respectively. In the presented method, no matrix effect was observed, where LOQ ranged between 1 and 5 ng mL^–1^ and LOD between 0.3 and 1.5 ng mL^–1^. Recovery ranged from 84.6 to 107.6% in oil and from 80.6 to 105.9% in marijuana. Precision was evaluated in three ways: within a single day (RSD: 3.14–10.87% in oil, 3.25–10.14% in marijuana), across different days with a second analyst (RSD: 1.98–10.71% in oil, 4.65–12.81% in marijuana), and laboratories with a third analyst (RSD: 5.59–13.94% in oil, 4.65–13.56% in marijuana). The proposed method offers high sensitivity, selectivity, and precision, being adequate and satisfactory for the quantification of CBD, CBN, CBC, CBDA, Δ^9^-THC, and Δ^9^-THCA.

## Graphical abstract

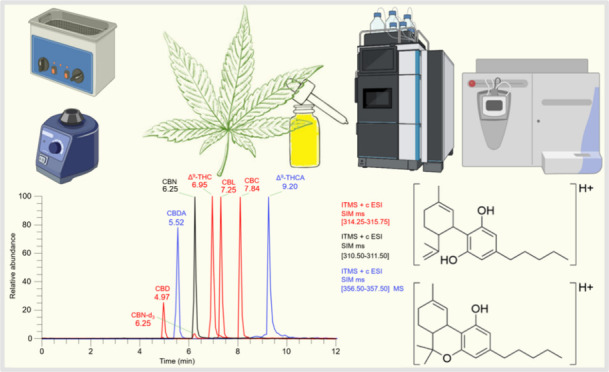

## Introduction

*Cannabis* refers to the genus of an angiosperm plant belonging to the *Cannabaceae* family, possessing a monotypic species named *Cannabis sativa* L., with possible variations such as *indica* Lam and *ruderalis*.^1^ This ancient species began cultivation approximately 12,000 years ago in China for fiber production. Over the years, its cultivation has been done for different purposes, such as medicinal use and as a drug, among others.^1,2^ Its wide applicability is attributed to its various constituents, with over 550 compounds identified, especially a class of terpenophenolic compounds known as cannabinoids.^1,3^

Cannabinoids or phytocannabinoids are a class of compounds unique to *Cannabis*, with 120 compounds identified to date, which can be divided into different groups: (1) Δ^9^-tetrahydrocannabinol (Δ^9^-THC), (2) Δ^8^-tetrahydrocannabinol (Δ^8^-THC), (3) cannabidiol (CBD), (4) cannabigerol (CBG), (5) cannabichromene (CBC), (6) cannabitriol (CBT), (7) cannabinodiol (CBND), (8) cannabielsoin (CBE), (9) cannabinol (CBN), and (10) cannabicyclol (CBL) and a group for various cannabinoids.^1,4^ Cannabinoids are predominantly present in their acidic form in the plant, being decarboxylated upon heating above 125 °C.^5^ Additionally, the main cannabinoids of interest are Δ^9^-THC, CBD, and CBN.^5^

Currently, *Cannabis sativa* L. is mainly cultivated for recreational use, followed by medicinal purposes.^6^ Among the cannabinoids, Δ^9^-THC is primarily responsible for the psychoactive effects of the plant, which is now the most trafficked drug according to the United Nations Office on Drugs and Crime (UNODC).^5^ Furthermore, the therapeutic use of cannabinoids is promising, with several treatments now available that utilize them, especially CBD and Δ^9^-THC.^5,6^ This application is possible due to the presence of endocannabinoids, neurotransmitters that bind to cannabinoid receptors present in the human metabolic system.^6^

In this context, due to the increased interest in *Cannabis* products, various instrumental analytical techniques are applied for the identification and quantification of cannabinoids. The most common include near-infrared spectroscopy (NIR), Raman spectroscopy, nuclear magnetic resonance (NMR), mass spectrometry (MS), and primarily chromatographic techniques such as gas chromatography coupled with mass spectrometry (GC-MS), gas chromatography with flame ionization detector (GC-FID), and liquid chromatography (LC) coupled with MS or spectrophotometric techniques.^4–8^

Considering these techniques, among the main ones, LC has the advantage of not requiring high temperatures, which prevents acidic cannabinoids from degrading during the process, unlike techniques such as GC-FID and GC-MS.^4,8^ Furthermore, when coupled with MS, it offers greater sensitivity and selectivity than spectrophotometric techniques.^4,8^ Finally, with advancements in modern chromatography, ultra-performance liquid chromatography (UPLC/UHPLC) allows process optimization, providing better separation and resolution with lower solvent consumption compared to high-performance liquid chromatography (HPLC).^9^

A review of the literature reveals numerous studies using HPLC and UHPLC, mainly coupled to spectrophotometric detection. However, many studies do not perform a complete validation of the methodology, fail to optimize chromatographic separation for various isomers, and rarely optimize the extraction process by evaluating different variables.^10–13^ Thus, the objective of this study was to evaluate the extraction of cannabinoids in oil and marijuana samples (pressed parts of the plant), along with the development and validation of a quantification method using UPLC-MS by ANVISA regulations governed by RDC No. 166, dated July 24, 2017.^14^

## Experimental Section

### Materials and Reagents

Δ^9^-THC, Δ^9^-THCA, CBD, CBDA, CBC, CBN, CBL, and cannabinol-d_3_ (CBN-*d*_3_) standards were purchased from Cerilliant (Round Rock, TX, United States of America). HPLC-grade methanol was obtained from Sigma-Aldrich (St. Louis, MO, United States of America). Formic acid was supplied by Honeywell (São Paulo, SP, Brazil). Water was purified using a Milli-Q purification system (Milford, MA, United States of America). Syringe filters with a 0.45 μm pore size were obtained from Analtica (Diadema, SP, Brazil).

### Sample Preparation

10 g of marijuana was freeze-dried for 12 h at −30 °C using an LS3000 freeze-dryer and ground, with a mortar and pestle, to obtain a homogeneous sample. 3 mg of the ground product and 20 mg of oil were extracted separately in 0.5 mL of solvent, filtered through a 0.45 μm filter, and diluted in methanol 1:9. An experimental design was performed using the Box-Behnken Design, where the extraction solvent (ethyl acetate, ethanol, and methanol), sonication time in minutes (10, 20, and 30), and sonication heating temperature in °C (40, 55, and 70), followed by vortex in minutes (5, 7.5, and 10) were evaluated. After optimization, the *Cannabis* extractions were performed as follows: 10 min in an ultrasonic bath at 70 °C, followed by vortexing for 7.5 min using ethyl acetate as the extraction solvent; for the oil, ethyl acetate was used to dilute the sample, then filtered and diluted again in methanol 1:9.

### Instrumentation

The UHPLC-MS analysis was performed on a Vanquish UHPLC system equipped with a Luna Omega C18 100 Å column (2.1 × 150 mm, 1.6 μm) with an Ultra C18 precolumn cartridge filter (2.1 mm 3/PCT). The chromatographic separation was performed using two phases: (A) 0.1% formic acid in ultrapure water and (B) 0.1% formic acid in methanol. The gradient elution occurred at a flow rate of 0.3 mL min^–1^, starting with 50% B increasing to 85% in 0.5 min, remaining constant for 4.3 min, and then rising to 94% in 1.7 min. Subsequently, the percentage of B increased to 100% in 0.5 min and remained constant for 2.8 min. Finally, the conditions were returned to the initial parameters in 0.0001 min and held for 2.2 min. The column temperature was maintained at 45 °C, and the injection volume was 5 μL.

For data acquisition, an LTQ XL Mass Spectrometer (Thermo Fisher Scientific, United States of America) with an electrospray ionization (ESI) source operating in both positive ESI­(+) and negative ESI(−) ionization modes was used. The ESI­(+) was employed for mass acquisition by selected ion monitoring (SIM) at *m*/*z* 311 and 315 ± 1, whereas ESI(−) was used for SIM at *m*/*z* 357. Finally, both ionization modes were also explored by using selected reaction monitoring (SRM) according to Table 1.

**Table 1:** Cannabinoids and *m/z* Were Monitored

cannabinoids	SIM (*m*/*z*)	SRM (*m*/*z*)
CBD/CBL/CBC/Δ^9^-THC	315	193, 235, and 259
Δ^9^-THCA/CBDA	357	313 and 359
CBN-d3	314	296
CBN	311	223 and 293

The ESI(−) mode was applied for mass acquisition by SIM with *m*/*z* 357 ± 1 and by SRM (Figure 1) according to Table 1. The ESI parameters were as follows: (i) capillary temperature: 350 °C; (ii) source temperature: 300 °C; (iii) sheath gas: 35 (arbitrary units); (iv) auxiliary gas: 10 (arbitrary units); and (v) source voltage: 4 kV (+) and 3.5 kV (−).

**Figure 1:**
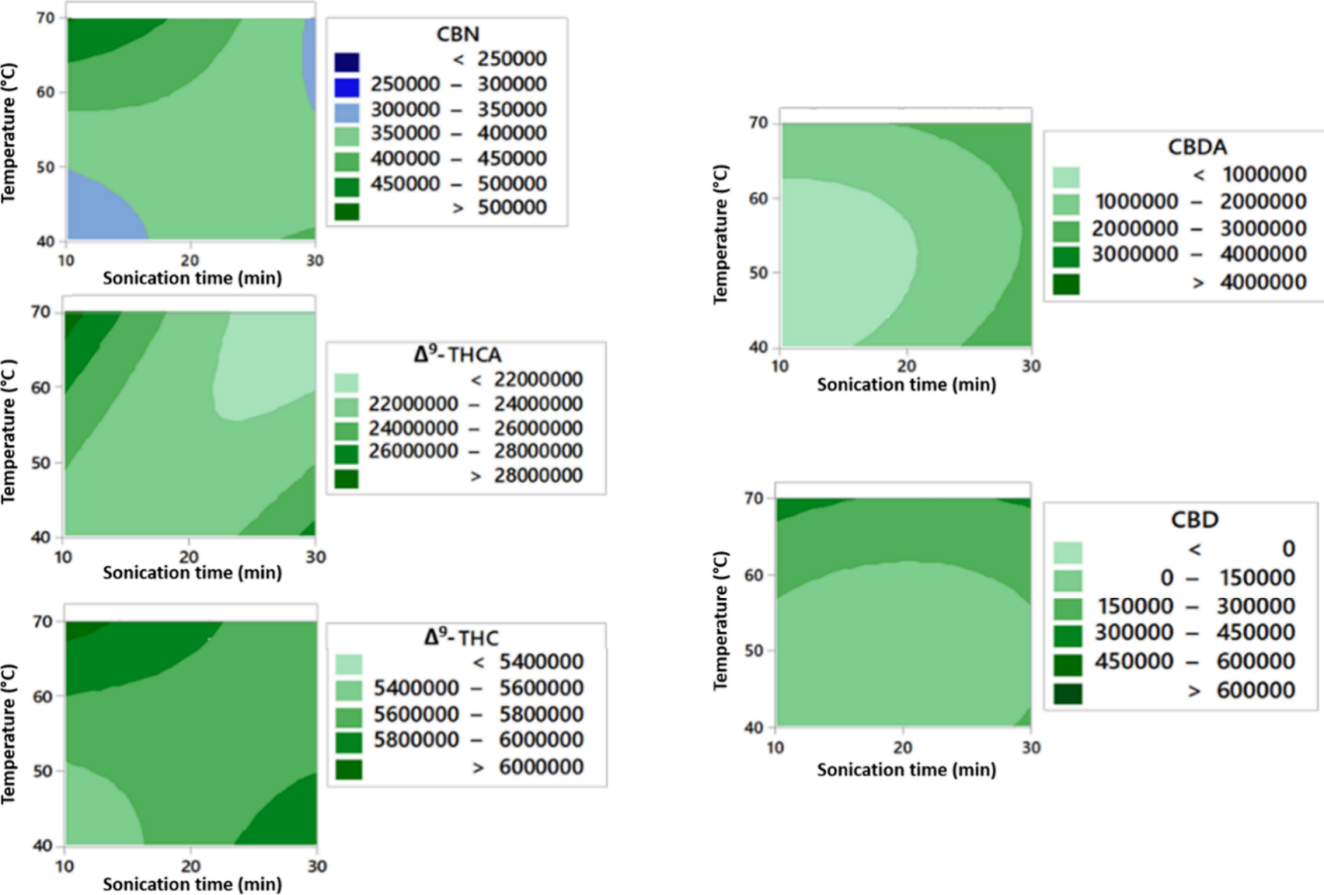
Contour plot of ultrasonic heating temperature in °C versus sonication time to the chromatographic peak area for Δ^9^-THC, CBD, Δ^9^-THCA, CBDA, and CBN cannabinoids.

### Validation

Matrix effect, working range, linearity, limit of detection (LOD), limit of quantification (LOQ), recovery, intermediate precision, reproducibility, and repeatability were evaluated according to the guidelines described by ANVISA, governed by RDC No. 166, dated July 24, 2017.^14^

The matrix effect was evaluated by constructing curves with 6 points in triplicate, adding analyte to the curve prepared in solvent and in the extracts diluted in methanol for both matrices. To overcome the matrix effect, CBN-d3 was added at 500 ng mL^–1^ as an internal standard in the final solution before injection. The curve was prepared by using concentration versus the area ratio of the chromatographic peak of the analyte and the *m*/*z* 296 fragment of the internal standard. For evaluation, the angular coefficients of the curves in solvent and matrix were compared statistically with the paired-sample *t;*test in Excel with α = 0.05. The LOD and LOQ were determined by the signal-to-noise ratio, with LOQ being the point where the signal is 10 times greater than the noise and LOD being the concentration where the signal is 3 times greater than the noise.

Linearity was evaluated using a six-point calibration curve and a blank for Δ^9^-THCA (blank, LOQ, 1500, 3500, 5500, 7500, and 9500 ng mL^–1^) and a seven-point curve and a blank in triplicate for the other cannabinoids (blank, LOQ, 1000, 2000, 3000, 4000, 5000, and 6000 ng mL^–1^). These values were selected to reflect all of the concentration ranges found in real samples, considering the proposed method. The curve was plotted using concentration and the area ratio of the chromatographic peak of the analyte and the fragment of *m*/*z* 296 of the internal standard at 500 ng mL^–1^.

For the recovery test, the assay was performed in triplicate with three different concentrations at three levels: low, medium, and high, within the curve points. For the repeatability study, six extractions were evaluated with the same analyst. For the intermediate precision study, six extractions were evaluated, varying the weeks of preparation and analysis along with the analyst. Finally, for the reproducibility evaluation, a third analyst in another laboratory performed the same analysis. The quantified cannabinoids were CBD, CBC, CBN, CBDA, Δ^9^-THC, and Δ^9^-THCA, and the presence of CBL in the chromatographic run was studied, where the separation of the seven cannabinoids was executed, with only the LOD of CBL being evaluated

## Results and Discussion

### Extraction

#### Extraction in Oil

The extraction optimization indicated that among the factors evaluated (solvent, sonication time, sonication temperature, and vortex time), only the solvent showed statistically significant differences between its tested levels, directly affecting the extraction efficiency (Table 2). Accordingly, the response optimizer (Figure S1) identified ethyl acetate as the condition that maximizes recovery, and it also enabled complete dilution of the oil with only 10 s of vortex mixing. Furthermore, during the dilution process, when the sample encountered the injection solvent, methanol, an emulsion was formed and quickly broken, allowing the separation of oil and solvent to be observed. However, the results showed that this process did not negatively affect the outcomes. As a result, ethyl acetate proved more efficient in comparison with ethanol and methanol as the extraction solvent. Therefore, the extraction process in oil was followed by solvent dilution, filtration, and dilution in methanol.

**Table 2:** Results of the Box-Behnken Analysis

parameters	oil	marijuana
solvent type	affected recovery	affected the recovery of CBN only
sonication time (min)	no significant effect	affected recovery
sonication temperature (°C)	no significant effect	affected recovery
vortex time (min)	no significant effect	no significant effect

Hsu et al. used methanol and sonication in the extraction of Δ^9^-THC, CBD, and CBN in cosmetic oils, requiring 30 min for the process. This time was necessary due to the use of methanol, which has difficulty solubilizing the oily sample, demonstrating an advantage of using ethyl acetate.^11^ In contrast, Palermiti et al. performed a preparation with dilution using 2-propanol to identify Δ^9^-THC, CBD, Δ^9^-THCA, and CBDA in oil. However, this required intense dilution of 1:20 oil/isopropanol, which could affect the final concentration of some cannabinoids, especially CBC, which was assessed in our study and is present in the matrix in very low concentrations. Additionally, the variation in cannabinoid content across different hybrids and samples may negatively affect the results when a significantly high dilution is applied.^15^

#### Extraction in Marijuana

The results obtained from the experimental design indicated that the difference between vortex agitation times was not statistically significant, allowing the use of any of the three variables evaluated (5, 7.5, and 10 min) (Table 2). To proceed with the study, the central point of the vortex agitation time was chosen. It is important to emphasize that combining an ultrasonic bath with vortex agitation is advantageous when working with plant material, as the ultrasonic bath breaks the cell walls, allowing greater contact with the solvent, enhanced by the addition of agitation.^16^

Regarding the solvent, a statistically significant difference was observed only for the extraction of CBN, for which ethyl acetate proved to be the most effective option. Figure S2 illustrates that ethyl acetate provided a higher recovery in this case.

Furthermore, when considering the extraction of the other cannabinoids and the toxicity profile of the solvents, ethyl acetate and ethanol emerge as more suitable alternatives than methanol.^17^ Moreover, in cannabinoid extraction, nonpolar solvents are more effective for extracting neutral cannabinoids but have low efficiency in extracting acidic cannabinoids.^16^ Thus, given that all of the solvents are polar, ethyl acetate demonstrated greater efficiency in the proposed extraction for CBN and was slightly less polar than the others, making it the chosen extraction solvent for marijuana.

The sonication time and temperature were evaluated according to the contour plot shown in Figure 1.

The contour plot depicting the relationship between the ultrasonic bath heating temperature and sonication time highlights the impact of the evaluated values on the extraction efficiency for each cannabinoid. Observing each cannabinoid individually, CBN achieved its optimal extraction point using 10 min of sonication at 70 °C, with a decline in efficiency at all other evaluated conditions. Similarly, Δ^9^-THCA reached maximum yield at 10 min of sonication at 70 °C. Conversely, CBDA showed optimal extraction efficiency at 30 min of sonication, with no significant changes observed at different temperatures.

For the cannabinoids of greatest interest, Δ^9^-THC and CBD, extraction efficiency was primarily influenced by 10 min of ultrasonic sonication at 70 °C. Alternatively, 30 min at 70 °C can be applied for better CBD extraction yields. Therefore, to maximize the extraction of the studied cannabinoids, a sonication time of 10 min at 70 °C was selected. Moreover, 70 °C proved to be more adequate for the extraction of the acidic cannabinoids, indicating that decarboxylation was not observed in the method.

Ultrasonic bath extraction has been widely applied for obtaining bioactive compounds, significantly reducing processing time compared to conventional methods such as maceration and Soxhlet extraction.^18–20^ Temperature and sonication time play crucial roles in the sonication process, with their effects being either positive or negative depending on the evaluated conditions.

The results indicate that high temperatures can optimize the extraction process but may also reduce recovery, depending on the applied sonication time. A temperature of 70 °C is satisfactory for shorter sonication times but becomes unsuitable for extended durations. This is likely due to the potential sample degradation during prolonged sonication. Additionally, the energy applied during the process may increase the temperature beyond the initially set value, leading to analyte degradation.^16,18^ Consequently, at 70 °C, 10 min of sonication resulted in efficient extraction with higher recovery rates.

Agarwal et al. studied the effects of power, sonication time, and solvent, finding that applied power did not significantly affect extraction yield. However, among the time variables, 15 min (the maximum tested) positively influenced the results.^18^ In contrast to the results presented here, where longer ultrasonic bath times increased yields, the study did not examine temperature effects, suggesting that higher temperatures might reduce ultrasonic bath extraction times.

#### Instrumentation

The chromatographic method was prepared to achieve adequate resolution for quantification. Various elution gradients were evaluated to ensure satisfactory separation of peaks within a reasonable time frame, considering the number of cannabinoids analyzed. The result, shown in Figure 2, demonstrates the successful separation of seven cannabinoids in a 12 min run. Finally, it is important to note that coeluting peaks with different *m*/*z* values, such as CBN and CBN-d_3_, can be effectively separated using the extracted ion chromatogram.

**Figure 2:**
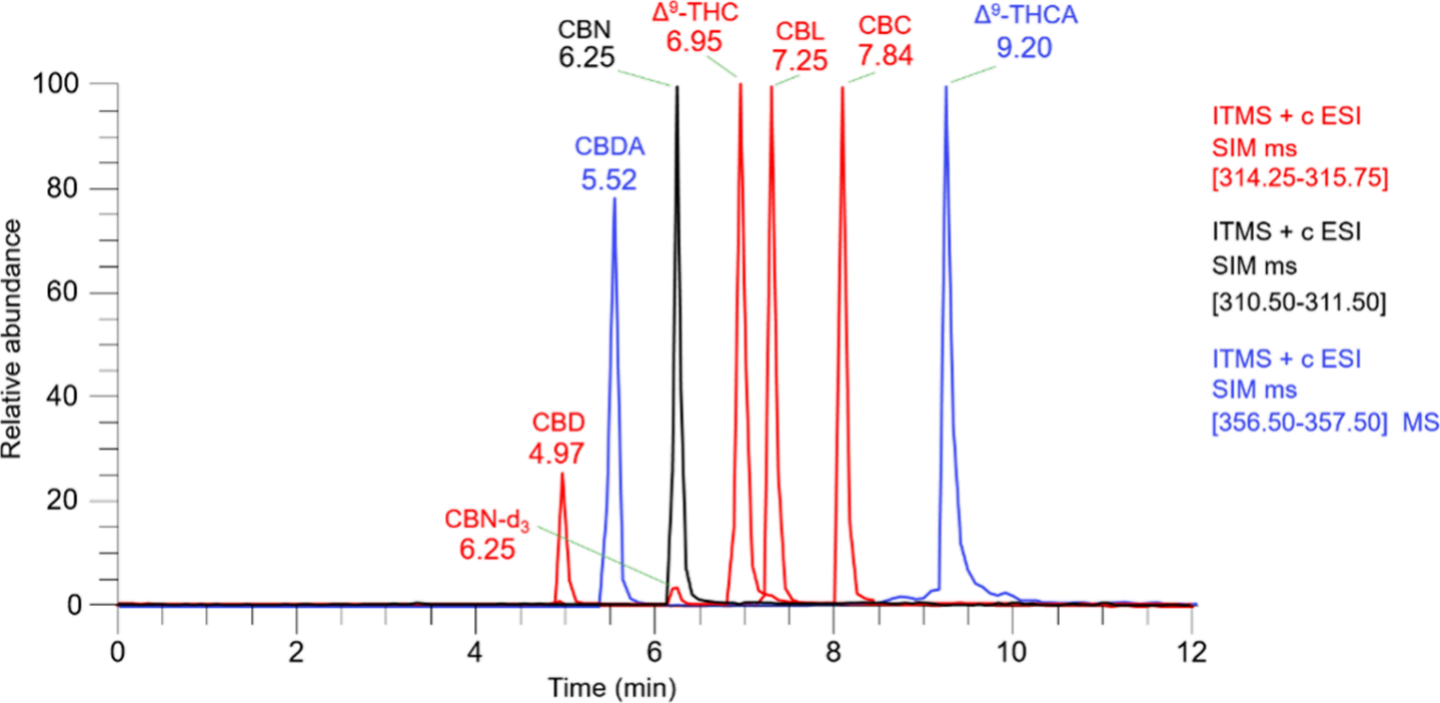
Chromatogram of analytes at 1000 ng mL^–1^.

According to Figure 2, the retention time pattern of the cannabinoids can be observed, where the elution order aligns with what has been previously reported in the literature.^21,22^ The separation results from the interaction between the stationary and mobile phases with the analytes, which possess distinct chemical structures and properties.^5,23^ Consequently, it is presumed that the CBD, CBDA, and CBN cannabinoids exhibit less interaction with this C18 column than Δ^9^-THC, CBL, CBC, and Δ^9^-THCA in relation to the proposed mobile phase.

Regarding chromatographic resolution, the separation between CBL and Δ^9^-THC was less prominent compared to other cannabinoids but still appropriate for quantification. Moreover, CBL (*m*/*z* 315) mainly fragments into *m*/*z* 235, and this signal can be used to differentiate it from Δ^9^-THC. Finally, this cannabinoid was not detected in the studied samples, and the separation, though minimal, remains sufficient for the quantification of both compounds. Concerning CBL levels in real samples, few studies address its identification and quantification, complicating this discussion. In this context, Turner et al. indicated that samples with higher CBC content generally also contain higher amounts of CBL, and the results from this study suggest that the CBC content in oil and plant samples is low.^24^

In this regard, Pate proposed that CBL is likely a degradation product of CBC, explaining its presence in the plant as being linked to high CBC concentrations.^25^ Glivar et al. analyzed the cannabinoid content in various industrial hemp samples from Slovenia and found no CBL in any of the samples, even when CBC was present.^26^ Furthermore, the CBC levels in the samples were significantly lower than those of other cannabinoids such as CBD and Δ^9^-THC. Considering the literature and the samples studied, it was not possible to quantify CBL and include it in the validation process. Its inclusion in the chromatographic run was intended to ensure the separation of Δ^9^-THC and confirm that its potential future presence would not interfere with the results.

Additionally, the parameters of UHPLC-MS were evaluated to achieve greater sensitivity and selectivity in the method. Figure 3 presents the extracted ion chromatogram at 1 μg/mL for both (**A**) ESI­(+) and (**B**) ESI(−) obtained from LTQ MS, where Figure 4 shows the mass spectra of the main fragments used for confirmation.

**Figure 3:**
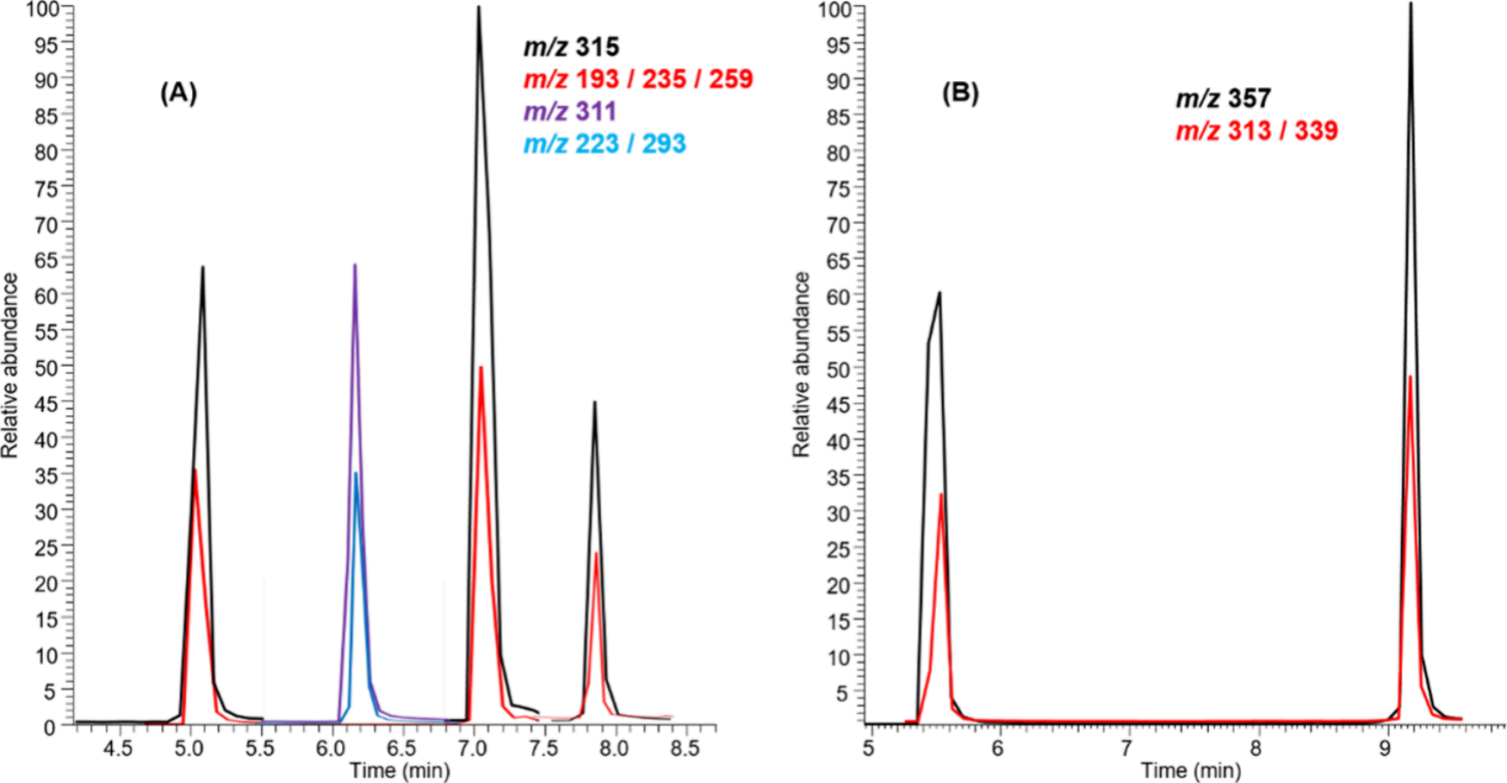
Intensity of monitored ions at 1000 ng mL^–1^. (A) ESI­(+) and (B) ESI(−) ionization modes.

**Figure 4:**
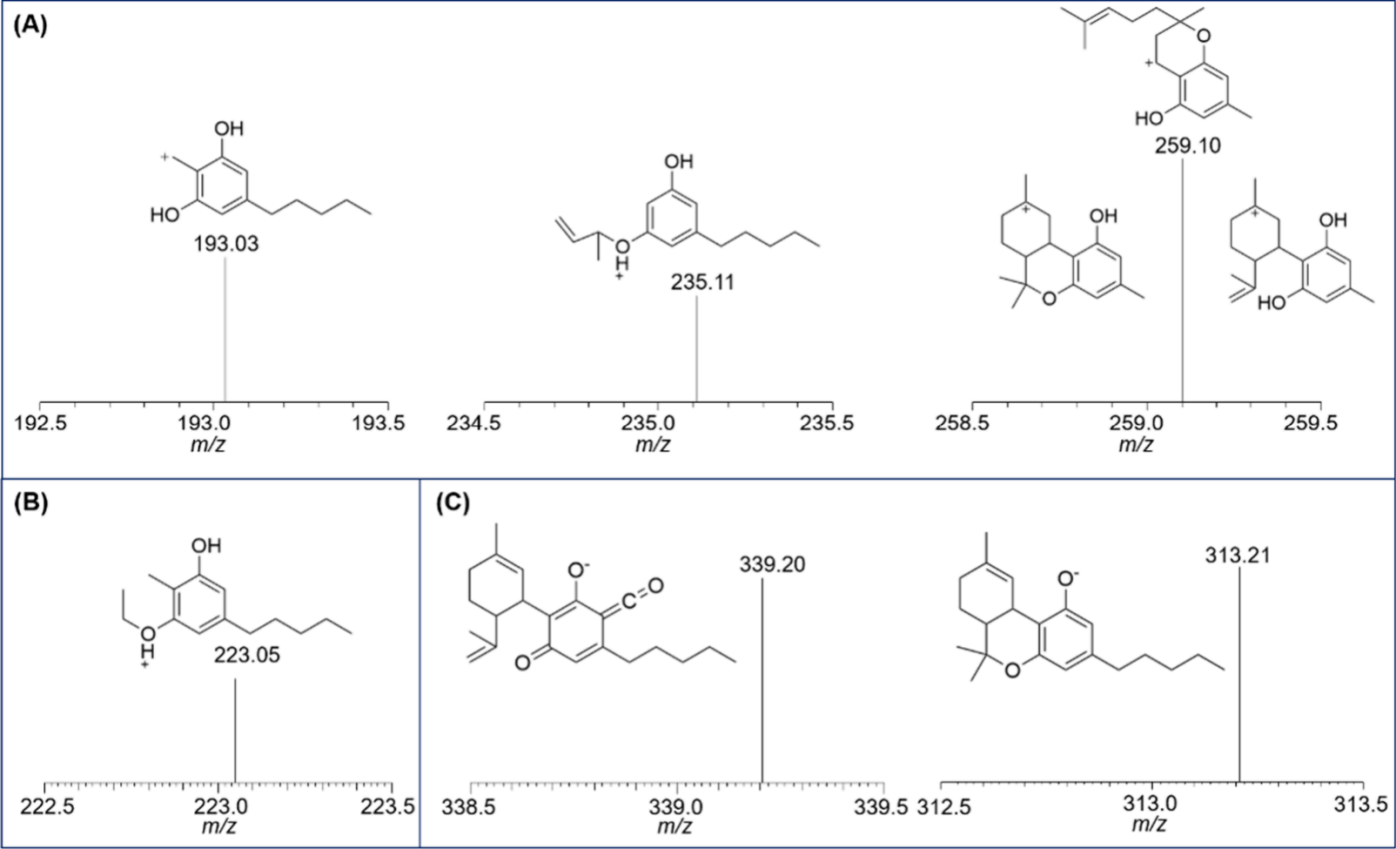
Mass spectra of the main fragments of the monitored ions. SRM transition for the precursor ion at (A) *m*/*z* 315 – ESI­(+), (B) *m*/*z* 311 – ESI­(+), and (C) *m*/*z* 357 – ESI(−).

According to Figure 3A,B, a decrease in fragment intensity relative to the precursor ion can be observed, which is expected since the collision energy did not completely dissociate the precursor. The SRM ions were selected based on the most intense and reproducible fragments of the monitored *m*/*z* values, ensuring reliable confirmation, given that cannabinoids exhibit different fragmentation behaviors. Although ion trap instruments are not commonly applied for cannabinoid quantification, the results obtained in this study demonstrated sufficient selectivity and robustness for the validated method.^27^

### Validation

#### Matrix Effect and Selectivity

The matrix effect study conducted without an internal standard indicated, by comparing the intersections of the curves, that the matrices studied can significantly suppress the signals observed with the proposed sample preparation. However, with the addition of the internal standard, the matrix effect was not observed and became statistically insignificant. Chromatograms of the samples show consistency of the method (Figure 5). Moreover, through this test, it was possible to confirm the selectivity of the method, considering that the presence of matrix interferences did not influence the result, especially with the use of the LTQ, which allows detection through the mass-to-charge ratio of the analyte and its fragments via MS/MS scans, making the method more selective.

**Figure 5:**
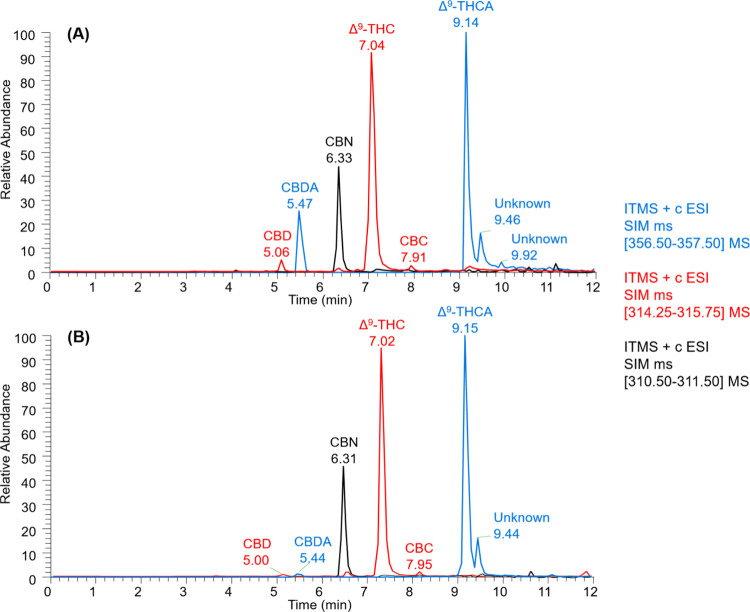
Chromatographic profiles of target cannabinoids in marijuana (A) and oil (B).

In this context, it is important to note that a more significant dilution in this case may result in the inability to detect some cannabinoids present at lower concentrations. Furthermore, according to the literature, it is difficult to find studies that investigate the matrix effect in *Cannabis* samples, which is a significant issue as this effect can influence the accuracy, precision, and sensitivity of the method.^28–30^

#### Linearity, Limit of Detection, and Quantification

Linearity was evaluated using the determination coefficient (*r* ^2^) and correlation coefficients (*r*). These parameters were applied for quantification in both oil and *Cannabis* samples. The linearity results, along with the LOD and LOQ, are presented in Table 3. The curve data were analyzed with Cochran’s test, confirming a homoscedastic system for all cannabinoids (Table S1).

**Table 3:** Determination and Correlation Coefficients, LOQ, and LOD

cannabinoids	***r*** ^2^	*r*	LOD (ng mL^–1^)	LOQ (ng mL^–1^)	equation
CBD	0.9962	0.9980	1.5	5.0	y=2.9488x−0.2726
CBC	0.9886	0.9943	1.5	5.0	y=1.0877x+0.1386
CBN	0.9968	0.9984	1.5	5.0	y=3.3415x−0.2094
CBDA	0.9819	0.9909	0.3	1.0	y=1.8852x+0.4195
Δ^9^-THC	0.9886	0.9943	0.3	1.0	y=3.169x+0.7844
Δ^9^-THCA	0.9949	0.9974	0.3	1.0	y=3.0789x+0.3913
CBL	-	-	0.3	-	-

The results for the LOQ and LOD were satisfactory and comparable to or below values reported in the literature, demonstrating high sensitivity when using a low-resolution MS such as the LTQ operating in SIM mode. Restricting the *m*/*z* range during acquisition in SIM mode enhances the greater method’s sensitivity. Thus, the narrower the mass range, the more specific the method, making it more suitable for quantification.^31^ Finally, for the *Cannabis* plant, a LOD of 0.05 and 0.25 mg kg^–1^ and LOQ of 0.16 and 0.8 mg kg^–1^ establish this method as highly sensitive.

#### Recovery and Precision

Given the lack of certified reference material for *Cannabis* samples, whether in oil or plant form, recovery tests were conducted with the cannabinoids present, and average results for each level are presented in Table 4. Recovery was calculated using eq 1:^14^

$$\mathrm{Recovery}\left(\%\right)=\left(\frac{C_{1}-C_{2}}{C_{3}}\right)\times 100$$ 1

**Table 4:** Recovery Values

cannabinoids	level	recovery (%) in oil	recovery (%) in marijuana
CBD	low	99.1	98.5
	medium	98.1	99.9
	high	100	103.1
CBC	low	107.6	99.1
	medium	92.5	90.5
	high	104	105.9
CBN	low	98.4	97.7
	medium	92.4	99.3
	high	97	103
CBDA	low	106.3	92.5
	medium	99.6	100.5
	high	109.2	103
Δ^9^-THC	low	103.9	92
	medium	94.8	93.2
	high	98	93.9
Δ^9^-THCA	low	98.1	83.4
	medium	90.8	80.6
	high	84.6	102.1

C1 is concentration of the analyte in the spiked sample; C2 is concentration of the analyte in the unspiked sample; and C3 is concentration of the analyte added to the spiked sample.

Table 4 shows that the average recovery ranged from 84.6 to 107.6% and 80.6 to 105.9% in oil and marijuana, respectively, complying with RDC No. 166, dated July 24, 2017 acceptance limits, and was comparable to or higher than values previously reported.^14,30,32–34^

The recovery assay is a crucial step, as the extraction process may lead to loss or incomplete recovery of the analyte.^32^ This can result in inaccurate estimations of the concentration of a compound of interest. Certified reference materials (CRMs), which are samples with known analyte concentrations, are commonly used in recovery studies. However, CRMs are not available for all products, such as *Cannabis* samples, in plant or oil form. To address this, the addition of a known analyte concentration to the sample enables the determination of the recovery percentage, ensuring accurate analyte quantification in the sample.^27^ Regarding the precision study, the results from the intralaboratory and interlaboratory tests are presented in Table 5.

**Table 5:** RSD Obtained in Precision Essays

cannabinoids	precision	Unit μg mL^–1^	RSD (*Cannabis* oil)	Unit μg mL^–1^	RSD (marijuana)
CBD	repeatability	0.10–1.00	4.29%	0.1–1	10.14%
	intermediate precision	0.10–1.00	3.27%	0.1–1	10.97%
	reproducibility	0.10–1.00	7.80%	0.10–1.00	10.69%
CBC	repeatability	0.10–1.00	8.45%	0.10–1.00	9.67%
	intermediate precision	0.10–1.00	10.71%	0.10–1.00	12.81%
	reproducibility	0.10–1.00	13.94%	0.10–1.00	13.56%
CBN	repeatability	0.10–1.00	3.14%	1.00–10.00	6.67%
	intermediate precision	0.10–1.00	5.24%	1.00–10.00	5.74%
	reproducibility	0.10–1.00	10.97%	1.00–10.00	7.06%
CBDA	repeatability	0.01–0.10	10.87%	1.00–10.00	9.84%
	intermediate precision	0.01–0.10	5.94%	1.00–10.00	12.56%
	reproducibility	0.01–0.10	8.45%	1.00–10.00	10.81%
Δ^9^-THC	repeatability	1.00–10.00	3.76%	10.00–20.00	3.25%
	intermediate precision	1.00–10.00	1.98%	10.00–20.00	5.01%
	reproducibility	1.00–10.00	9.36%	10.00–20.00	5.54%
Δ^9^-THCA	repeatability	1.00–10.00	3.93%	10.00–30.00	4.67%
	intermediate precision	1.00–10.00	5.98%	10.00–30.00	4.65%
	reproducibility	1.00–10.00	5.59%	10.00–30.00	4.65%

The guidelines followed indicate the acceptable DPR values for the different values found, with the unit being μg mL^–1^ or mg kg^–1^, and the regulation presents the maximum appropriate RSD value.^14^ In this sense, considering the results obtained, the maximum indicated RSD (%) values for the units of 100 ng mL^–1^ and 1, 10, and 100 μg mL^–1^ are 15, 11, 7.3, and 5.3%, respectively, with the concentrations found varying within these units as presented in Table 4. Moreover, few studies report full validation, including interlaboratory reproducibility, particularly in the context of *Cannabis* analysis in both oil and raw plant material.

Therefore, it is possible to conclude that the values found were satisfactory, meeting the requirements imposed by the regulations followed in this validation. Consequently, the method presented proved to be precise even when varying analysts, days, and laboratories. In this regard, considering that many studies in the literature do not simultaneously assess reproducibility and intermediate precision for these analytes, the results presented offer a higher degree of certainty regarding the consistency of the results and their variations.^32–34^

## Conclusions

The authors proposed an effective method for the quantification of cannabinoids in plant and oily samples, with the potential to be reproduced in different laboratories and by different analysts. The extraction demonstrated a satisfactory yield for all of the cannabinoids evaluated, standing out for its speed and simplicity across the different matrices analyzed. Additionally, liquid chromatography coupled with mass spectrometry proved to be an exceptional technique for quantitative cannabinoid analysis, offering high sensitivity and selectivity. These results not only contribute to the advancement of analytical methodologies for cannabinoid quantification but also establish a solid foundation for future research in the field.

## Supplementary Material



## Notes

Chemical structures, precursor ions and fragments, response optimizers (PDF)
