# Supplementary material for: Development and Validation of a Cannabinoid Quantification Method in Oil and Marijuana by UHPLC-MS
Source: ACS Omega. 2025 Nov 18;10(47):57385–93. doi: 10.1021/acsomega.5c07689 (PMC12676500; doi:10.1021/acsomega.5c07689)
Supplement: Supplementary file 1 [file ao5c07689_si_001.pdf]

Supporting Information

Development and validation of a cannabinoid quantification method in oil and marijuana by UHPLC-MS

João Victor M. de Almeida[a,b], Nathália S. Conceição[a,b], Alan Reinke Pereira[a,b,] Marcos Valério V. Lyrio[a], Rafael S. Ortiz[b,c], Nayara A. dos Santos[a,b,d], Wanderson Romão[a,b,d*]

[a]Federal University of Espírito Santo, Chemistry Department, 29075-910, Vitória-ES, Brazil.

[b]Instituto Nacional de Ciência e Tecnologia Forense (INCT Forense), Brazil.

[c]Brazilian Federal Police, Regional Superintendence of Rio Grande do Sul, 90160-093, Porto Alegre-RS, Brazil.

[d]Federal Institute of Espírito Santo, 29106-010, Vila Velha-ES, Brazil.

Correspondence should be addressed to: Wanderson Romão (wanderson.romao@ifes.edu.br)

Table of Contents

Figure S1. Solvent's response optimizer in oil extraction of all cannabinoids ((-1.0) methanol, (0) ethanol, and (1.0) ethyl acetate). The blue dashed line represents the optimal response value estimated by the model.
Figure S2. Solvent's response optimizer in marijuana of CBN. (-1.0) methanol, (0) ethanol, and (1.0) ethyl acetate. The blue dashed line represents the optimal response value estimated by the model.
Table S1. Cochran test results.

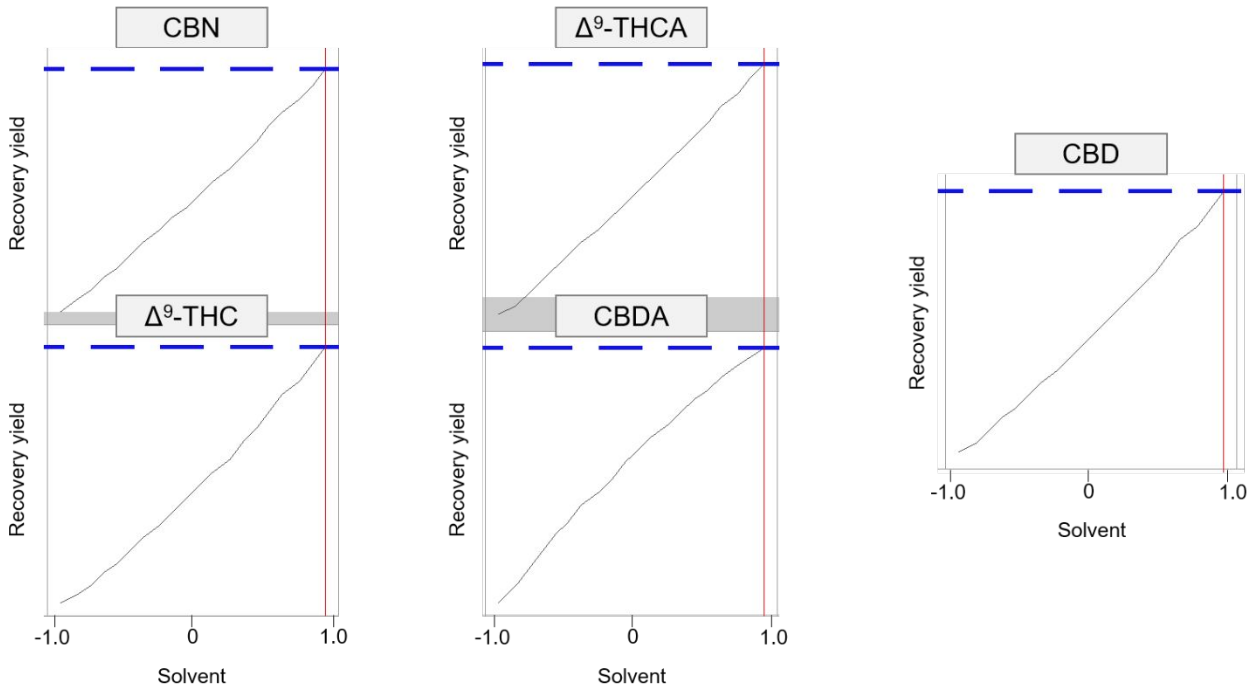

Figure S1. Solvent's response optimizer in oil extraction of all cannabinoids ((-1.0) methanol, (0) ethanol, and (1.0) ethyl acetate). The blue dashed line represents the optimal response value estimated by the model.

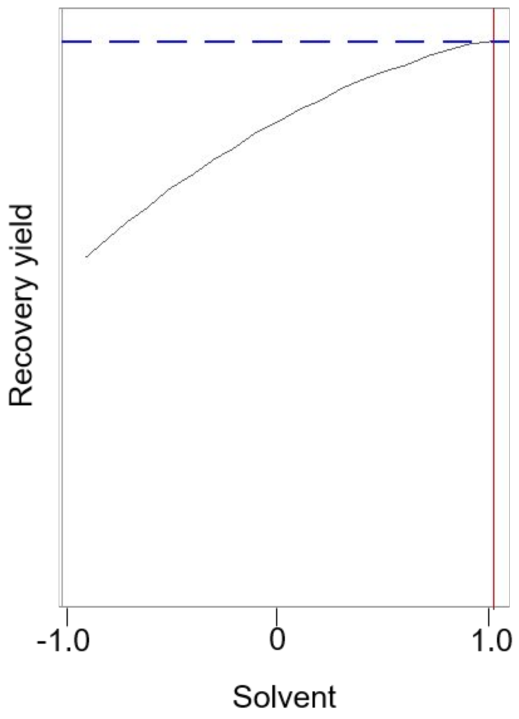

Figure S2. Solvent's response optimizer in marijuana of CBN. (-1.0) methanol, (0) ethanol, and (1.0) ethyl acetate. The blue dashed line represents the optimal response value estimated by the model.

Table S1. Cochran test results.

Cannabinoids	Calculated C	C critical
CBD	0,398	0,561
Δ^9-THC	0,285	0,561
Δ^9-THCA	0,423	0,516
CBDA	0,446	0,561
CBN	0,361	0,561
CBC	0,343	0,561
